# Vita activa in biotechnology: what we do with fungi and what fungi do with us

**DOI:** 10.1186/s40694-017-0041-2

**Published:** 2017-12-20

**Authors:** Martin Weinhold, Edeltraud Mast-Gerlach, Vera Meyer

**Affiliations:** 1 Menschenfotograf, Am Goldmannpark 21, 12587 Berlin, Germany; 2 Department of Applied and Molecular Microbiology, Institute of Biotechnology, Technische Universität Berlin, Gustav-Meyer-Allee 25, 13355 Berlin, Germany

**Keywords:** Innovative Training Network (ITN), Marie Curie, Hannah Arendt, Fungal biotechnology, QuantFung, Natural product, Secondary metabolite, Systems biology, Synthetic biology, EUROFUNG

## Abstract

Filamentous fungi are fascinating microorganisms. One of the reasons why it is so worthwhile to take a closer look at them is their capacity to produce secondary metabolites. Some of these substances have the potential to be of great use for mankind, such as it was the case with penicillin and its discovery in 1928. Almost a century later, the situation in healthcare could possibly turn back to the state before the development of the first antibiotics. Due to an overuse of antibiotics we are facing a surge of multiresistant bacteria that are not inhibited by any of the currently known drugs. That was part of the background why a European research project was launched in October 2013, titled “Quantitative Biology for Fungal Secondary Metabolite Producers”, or “QuantFung”. Fifteen young scientists embarked on a new phase in their career, moving to new work environments within Europe and dedicating their work lives intensively to the quest for useful secondary metabolites. After 4 years, the QuantFung project concluded in October this year. In this commentary, we aim to convey what it means to work in this field of fungal biotechnology and how important it is to improve the efficiency of the research therein. We introduce five out of the fifteen fellows at length and let them have their say about the adventure of science, euphoric moments, prospects and doubts. We also raise questions about the current state of research in academia, something the QuantFung fellows experienced first-hand. Being a scientist often goes beyond earning money to make one’s living. This is why we also reflect on aspects of the meaning of work in our western society, where production for profit’s sake is a main driver. For that we refer to one of the most distinguished thinkers of the twentieth century, to Hannah Arendt.

## Background

In 1960, political theorist Hannah Arendt chose “Vita activa” as the title for the German edition of her ground-breaking book “The Human Condition”, a highly complex text elaborating on what we actually do when we work [1]. “Vita activa” as a philosophical term means to participate socially in the complex structures humans create to organize themselves. Arendt was highly in favour of using the precious good of a given lifetime for the real capacities that humans possess. Applied to today’s world of work, it would be about capacities that go way beyond the daily toil which is necessary to make ends meet. In a job-focused society, where income often matters more than input, it may sound demanding to ask for a truly meaningful work life. Though closely observing recent phenomena in the realms of science or industry, it shows that we are limiting our perspectives when narrowing them down to profit margins. In one example, as it was more profitable, the German car industry was investing in new software to deceive testing methods for exhaust fumes instead of developing new technologies that would make the era of internal combustion engines a historic one altogether. Turning towards the antibiotics market: since the rewards are too low, the number of pharmaceutical companies worldwide went down from 18 in 1990 to 4 in 2010 [2]. Having the current crisis of antibiotic resistance in mind, that is threatening news.

In October 2017, a European research project concluded that produced a network for 15 young researchers working in five different European countries. The project started in October 2013 as a 4-year multi-partner Innovative Training Network (ITN), as part of the Marie-Curie Actions in the Seventh Framework Programme ‘People’ (FP7 people) and was provided with €3.9 million (EU Grant number 607332). The focus of the “QuantFung” project was to identify and isolate novel bioactive molecules from filamentous fungi. One of the triggers for launching this 4-year project was the crisis of antibiotic resistance. QuantFung was offering what should be a matter of course for such an important field of science: an efficient, result-based network of researchers with diverse backgrounds and expertise, where communication can flow freely and no efforts are doubled. Even though the work of these 15 highly committed researchers took into account that finally their new knowledge may result in new products for the market, it was not its driving force. Motivations to join QuantFung were manifold. For all participants the chance to gain new knowledge in a fascinating field of research was compelling—“unlocking (a) secret door”, as Min Jin Kwon, one of the researchers put it [3]. Nonetheless, the idea of producing something that society can really benefit from, is of major importance for them. Referring back to Hannah Arends idea of a meaningful life, in which humans can unlock their own capacities and talents, working for the QuantFung project truly qualifies for that. In this paper, we take stock of what was achieved within the framework of this exciting project, and also shed light on life models in (fungal) biotechnology. Kindly, a number of QuantFung fellows agreed to give a glimpse of what their life was like during the last 4 years and what they think can be learnt from their experience for future research projects.

## The origins of QuantFung

The quest for novel bioactive fungal products goes far beyond the problem of the antibiotics crisis, as it includes the need for novel drugs for multiple human health problems ranging from different cancers to increasing neurodegenerative diseases, which are especially prominent in aging societies. The necessity for a multidisciplinary training network that can unlock the genomic potential of filamentous fungi to produce novel bioactive compounds for human welfare was identified at a meeting of the EUROFUNG consortium in November 2010. EUROFUNG is a virtual centre of excellence and includes 35 academic members from 13 European countries and an associated industrial platform of 9 small, medium-sized and large European biotechnological and pharmaceutical companies [4]. A cross sector group of EUROFUNG active in the field of fungal bioactive compounds developed the initial idea for the QuantFung project (Technische Universität Berlin, Universität Göttingen, Danmarks Tekniske Universitet, DSM). QuantFung stands for “Quantitative Biology for Fungal Secondary Metabolite Producers”. The concept was further developed at the ESF-EMBO symposium “Synthetic biology of antibiotic production” in October 2011 [5], where additional groups joined (Rijksuniversiteit Groningen, Leibniz-Institut für Naturstoff-Forschung und Infektionsbiologie Jena—Hans-Knöll-Institut, Chalmers tekniska högskola, Debreceni Egyetem, Christian-Albrechts-Universität zu Kiel, Bioviotica Naturstoffe, HiTeXacoat, Planton GmbH, Codexis Laboratories Hungary LTD). The intended mission of the ITN was to combine internationally renowned research experience of the EUROFUNG members with new groups and their expertise in new technologies for a training network exploring the potential of fungi for innovative, bioactive products for biotechnology and, at the same time, training high quality researchers. This intention resulted in a first proposal submitted in January 2012 that scored 93.0 points and was placed on the EU reserve list for funding. A revised second proposal had been improved by addressing the helpful and constructive comments from the reviewers of the first evaluation. It also benefited from new ideas and refinements that came from a two-day workshop in October 2012 in Berlin. The submission of the subsequent proposal happened in November 2012 and it was approved in April 2013 with a score of 93.2.

After negotiations involving all academic and industrial partners, the QuantFung project eventually started in October 2013 as the first European training platform for the production of novel bioactive compounds based on fungal systems and synthetic biology [6]. Six months were devoted to selecting and recruiting the best fellows among the applicants. The eventual start-up meeting for all research projects within QuantFung was in March 2014, which was nearly 3.5 years after the initial idea for this EU project was launched.

## Combining excellence in training and research

The driving force of QuantFung was the collaboration of 8 academic and 5 industrial partners to expedite the application of new secondary metabolites in areas such as health care, nutrition or agriculture. The main objectives of the consortium were to find novel bioactive molecules by exploiting the wealth of fungal biodiversity and to translate these into useful products. This required the (re-)design and engineering of fungal organisms with new characteristics, using highly sophisticated synthetic biology tools. The educational idea of QuantFung was to use this research context to train 11 Ph.D. students and 4 Post-Docs as new problem-solving, creative European scientists in interdisciplinary and intersectorial biotechnological research. The fields varied from modelling and network analysis to systems biology (e.g. genomics, transcriptomics, proteomics and metabolomics), from molecular biology, like fungal genetics and biochemistry, to synthetic biology methods. Different work packages were designed which focused on discovery of secondary metabolite gene clusters, targeted activation of gene clusters, quantification of secondary metabolites in industrial hosts, and bioactivity testing to identify their mode of action. In order to bridge the gap between academia and industry, the industrial partners offered secondments and training modules to share their experience with the trainees. As these multidisciplinary projects required physical and intellectual flexibility, the training program for the young fellows included defined work periods in different QuantFung laboratories, as well as local and networked training events for researchers. The vision of the QuantFung partners was that such an approach would promote the development of a new generation of fungal biotechnologist with experience in both the academic and the industrial work culture, comprising significant translational and entrepreneurial skills.

## What has been achieved?

We decided to address this question on two different levels: On the one hand, we gathered all relevant data and information regarding the scientific output of QuantFung (e.g. publications, patent applications, poster presentations, talks at conferences). On the other hand, we were using interviews to discuss with members of the consortium the impact QuantFung had on their personal life and personal development.

Regarding the evaluation of “hard facts”, we took the project’s official end as an effective end date (October 1st, 2017), even with the knowledge that much of QuantFung’s output cannot yet be taken into account as many manuscripts are still in preparation or have just been submitted and are currently under review. Therefore, the assessment of the endeavor’s effectiveness and outcome can currently only be a partial one. The overall impact will be most likely revealed in 1 or 2 years’ time from now. However, the fellows’ accomplishments are already very impressive at this early stage: On average, each fellow presented his/her work at four (inter)national conferences and has been co-author of 1.9 publications (Table 1). Three of these publications have receive particular attention by the research community: (1) the establishment of a CRISPR/Cas9 based genome editing tool for *Penicillium chrysogenum* [7], (2) the sequencing of nine different *Penicillium* genomes and the identification of 1317 putative secondary metabolite gene clusters hidden there [8], (3) the establishment of polycistronic gene expression in the cell factory *Aspergillus niger* as a tool for high level production of secondary metabolites [9]. As a team, the QuantFung participants jointly organised one Mini-symposium in 2014 during the annual meeting of the German Society of General and Applied Microbiology (VAAM) and a session during the highly prestigious Conference on the Physiology of Yeast and Filamentous Fungi 2016 in Lisbon (PYFF6). The fellows used the opportunity to publicize QuantFung to the broader audience of biotechnologists and the interested public, and collectively co-wrote a commentary piece in 2015 [3], which resulted in over 2590 downloads during the first 2 years after its publication.

**Table 1:** Dissemination of QuantFung results (referring only to publications, patents or presentations within the 4-years funding period)

	Co-author of peer-reviewed publications^b^	Co-author of patent applications	Presentations at conferences (poster or talk)	Future perspectives/remark
Ph.D.^a^				
1	2	–	8	Successfully defended Ph.D. and continues research career as Post-Doc in academia working on fungi
2	1	–	8	Ph.D. defence date is envisaged, currently in job interviews
3	3	2	4	Ph.D. project continues for a 4th year (not funded by QuantFung)
4	1	–	4	Ph.D. project continues for a 4th year (not funded by QuantFung)
5	3	–	5	Ph.D. defence date is envisaged, currently in job interviews with academia
6	2	–	4	Ph.D. project continues for a 4th year (not funded by QuantFung). Already obtained a permanent position in a University to work on fungi as a fermentation engineer
7	1	–	2	Ph.D. project continues, which is however not funded by QuantFung
8	2	–	4	Ph.D. defence date is envisaged, currently in job interviews with industry
9	4	–	4	Ph.D. project continues (not funded by QuantFung)
10	1	–	–	Ph.D. project continues for a 4th year (not funded by QuantFung)
11	1	–	3	Ph.D. project continues for a 4th year (not funded by QuantFung)
Post-Doc^a^				
1	1	–	7	Continues career in a private-public cooperation project at an University working on fungi
2	1	–	3	Continues career in industry
3	3	2	3	Obtained an Assistant Professorship position
4	2	–	2	Obtained a permanent position at a research institution

^a^Note that projects funded by QuantFung for Ph.D. students were for 36 months, whereas post-doctoral fellows were funded for 24 months only

^b^Note that one publication was a joined publication of all fellows [2]

On paper, these years of research on filamentous fungi were very successful. But what impact did this time have on the fellows? We focused on five fellows who provided a personal perspective—Min Jin Kwon, Sietske Grijseels, Yvonne Nygård, Carsten Pohl and Jens Christian Frøslev Nielsen.

### The lab away from home

An essential part of the QuantFung ITN project was its mobility rule: the fellows had to move to a European country where they had not lived during the past 3 years.

For Yvonne, one of the experienced researchers in the group, it meant going from Finland to the Netherlands. As she is used to being much further away for work assignments (e.g. being for some time in Berkeley, USA, as a visiting researcher), the distance between her home country and the Netherlands was not a major issue. Going for visits back to Finland on an average of every 2 months proved to be sufficient, and convenient due to non-stop flights from Amsterdam to Helsinki. In Yvonne’s perception the main cultural difference was the very direct way of Dutch communication, although in the lab Dutch people actually were a minority, as she says, there it was way more international.

Min Jin, another of the experienced researchers, moved from the Netherlands to Berlin to participate in the project. It was a familiar place for her, as she had come to the German capital before for her master’s degree after graduating in South Korea where she was born. Min Jin had lived and worked for 5 years straight in the Netherlands before QuantFung, therefore she was qualifying for the mobility rule.

For Sietske, Carsten and Jens their respective change of country kept them culturally fairly close to what they knew from home. There were no greater “shocks” to cope with than maybe sweeter pastry for Sietske in Denmark or for Jens the Swedish consensus culture which was quite different from what he was used to in Denmark, he explains in the interview. Carsten even said about his time in Groningen (Netherlands) that he sometimes felt as if he were just in another German city. “But where I really felt like being in Europe was in my lab. There was the world at home.” he continues. Counting all the nationalities he was working with in the lab actually takes him a while, “…that was like a world community, you can surely say that”. The international aspect of their research environment—and its positive effect—is a shared perception among the QuantFung fellows. As Jens mentions about his group in Gothenburg: “The main nationality here is not Swedish, it’s Chinese. Then you have Indian, quite a few Germans and so on. So it’s not only the new Swedish culture I learnt about, it’s about all other cultures from just anywhere in the world.”

Nonetheless there are challenges when working abroad. For instance, figuring out things like tax authorities and insurances, which at the same time trains your ability for trouble-shooting and asking colleagues who have been in the same situation. Then there were the long commutes when you wanted to see family and friends during a weekend, using up most of the Friday for travelling. Even seemingly close distances within Europe take a toll and, as it was also about getting to know the other country and the people there, travel back home did not occur every weekend. For Jens it is one of the reasons why he will look for employment in his home country Denmark after the QuantFung project. His overall balance with the project is absolutely positive but he wants to be back where family and friends are. “This kind of work life-style (like during an ITN project) does come at a cost” he says. Whereas for Sietske going back to the Netherlands would now feel a bit like going back to a previous life. After the project she will move to Tromsø in Norway instead, for living there with somebody she got to know during her time in Denmark. “In that way you could say it’s an outcome of the QuantFung project.” she says with a laugh.

### The balance between work and private life

According to Hannah Arendt’s *Vita activa* (*The Human Condition*), excellence can only be achieved in the public realm, as this is the place where we can prove ourselves as being one of the best, as the place where we can stand out because there is comparison and because there are people who will notice our achievements. Nothing humans ever do in private can achieve this potential. Nonetheless there is the need to have a retreat where we are not seen and heard all the time and can recover [1]. The QuantFung fellows certainly moved onto a public stage when entering the international research community during the project. How did they perceive the balance between their work and the private life?

**Fig. 1:**
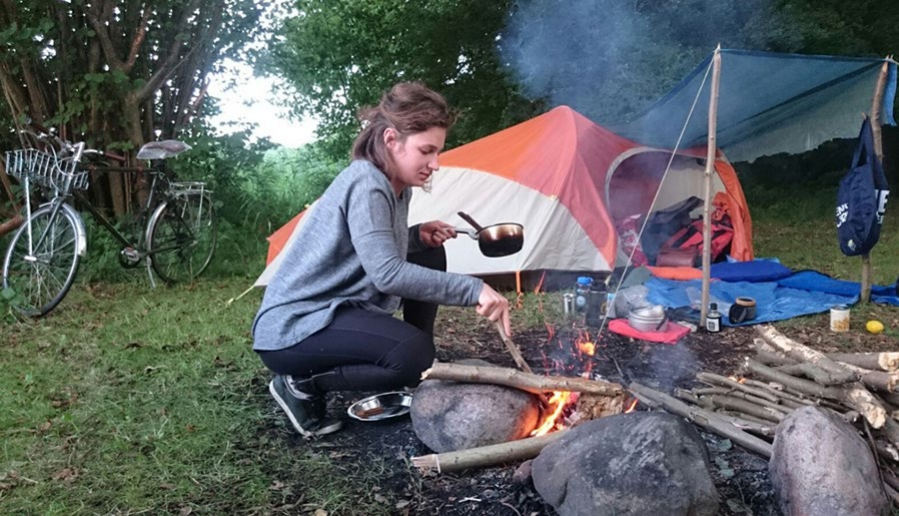
Sietske during a summer weekend trip to Denmark’s largest lake Arresø (© by Aaron Andersen)

Sietske (Fig. 1) is looking back very happily with what she experienced in Denmark at DTU. There was spare time, there were weekends for going out by bike for camping tours, which she loves doing. There was also a life besides fungi. However, considering future work options for herself she tends towards favoring a job in industry, despite the fact that she really likes her field of research and enjoys being at a university. There is the difference between her work life during QuantFung, where it was totally okay to go home after 5 pm, and then there is the other side of academia that she observed: “When I see the professors around here at DTU, many of them have a family but they work day and night to do the research and to apply for grants at the same time.” Especially for starting a family it could be very hard, she thinks “You find a post-doc for one or two years and during that period you already have to start writing grant applications or applying for new positions. … It’s really insecure and it’s not sure whether you can stay in the same country. I think it could be pretty hard to start a family in such a position.”

**Fig. 2:**
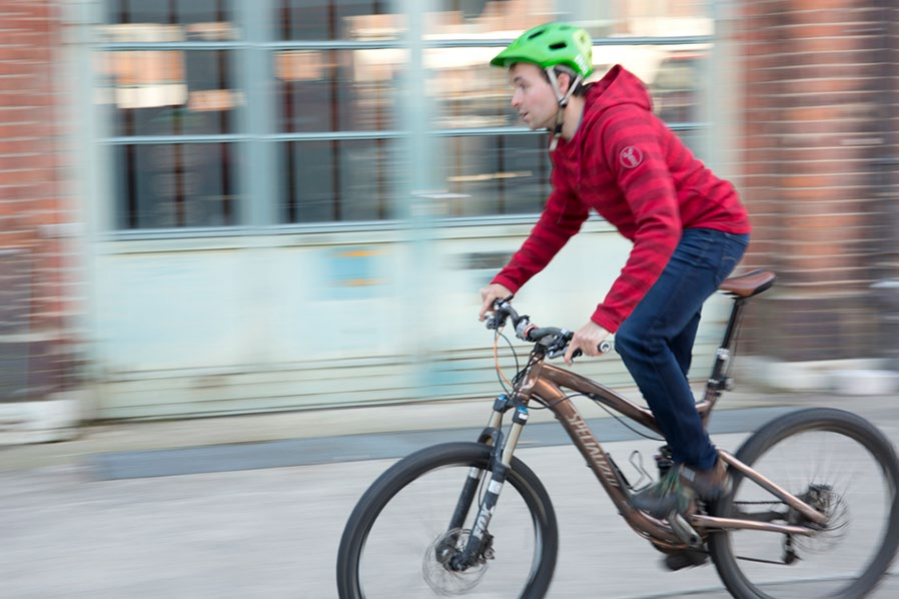
Testing the European mobility rule: Carsten racing to the interview appointment in Berlin, 2017

In the project’s beginning, Carsten (Fig. 2) was involved in sporting activities outside work, like fencing classes, “…but then there was such a dynamics that literally sucked me in. One thing led to another and it became more and more exciting and then you were checking the clock and it was 9 pm and you said to yourself, okay now it really is too late for exercise…” For a tangible break he needs a change of location, as work issues remain too preoccupying for as long as he is close to the lab: “I don’t go home in Groningen, close my door and then I don’t think about my work anymore, that’s nothing I am capable of doing. … There was an intense phase during the project in which I actually didn’t see the people at home, in my flat-sharing community, at all.” Carsten sees it also as a way to find a personal limit with work. He says it is mainly your own responsibility to decide how much you let yourself being consumed personally through work. “For me it was voluntary, I really enjoyed that and it was not done under pressure and force at all. … But it’s not a life model that works as a permanent set-up, certainly not.” During longer vacations that brought him away from Groningen and the lab, Carsten participated for instance twice in a huge bicycle race at the Nürburgring (Rad am Ring), something that required proper preparation and therefore was a real change of subject.

**Fig. 3:**
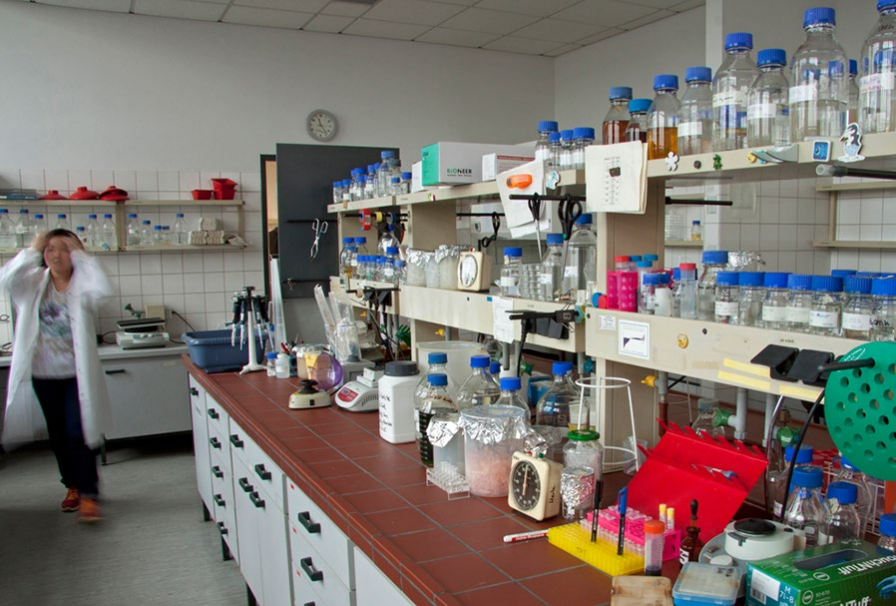
Min Jin entering one of the labs of the TU Berlin, 2015

Min Jin (Fig. 3) was living with her family in Berlin and therefore combined being a mother of two kids and pursuing the project’s ambitious research goals. Within the project’s run she took a 10-months maternity leave after giving birth to her second child. That is why she says without hesitation: “Within the QuantFung project there definitely was a good balance for me between private and work life.” The favourite thing besides the kingdom of fungi for her is playing with her children. But during the 6 months in which she had to come to the lab late at night for fostering demanding fungal bioreactor cultivations, she discovered Harry Potter novels as a diverting read. “Whenever I was going by public transportation at night or in the early morning, I appreciated J.K. Rowling, as she made me forget all the tiredness.”

**Fig. 4:**
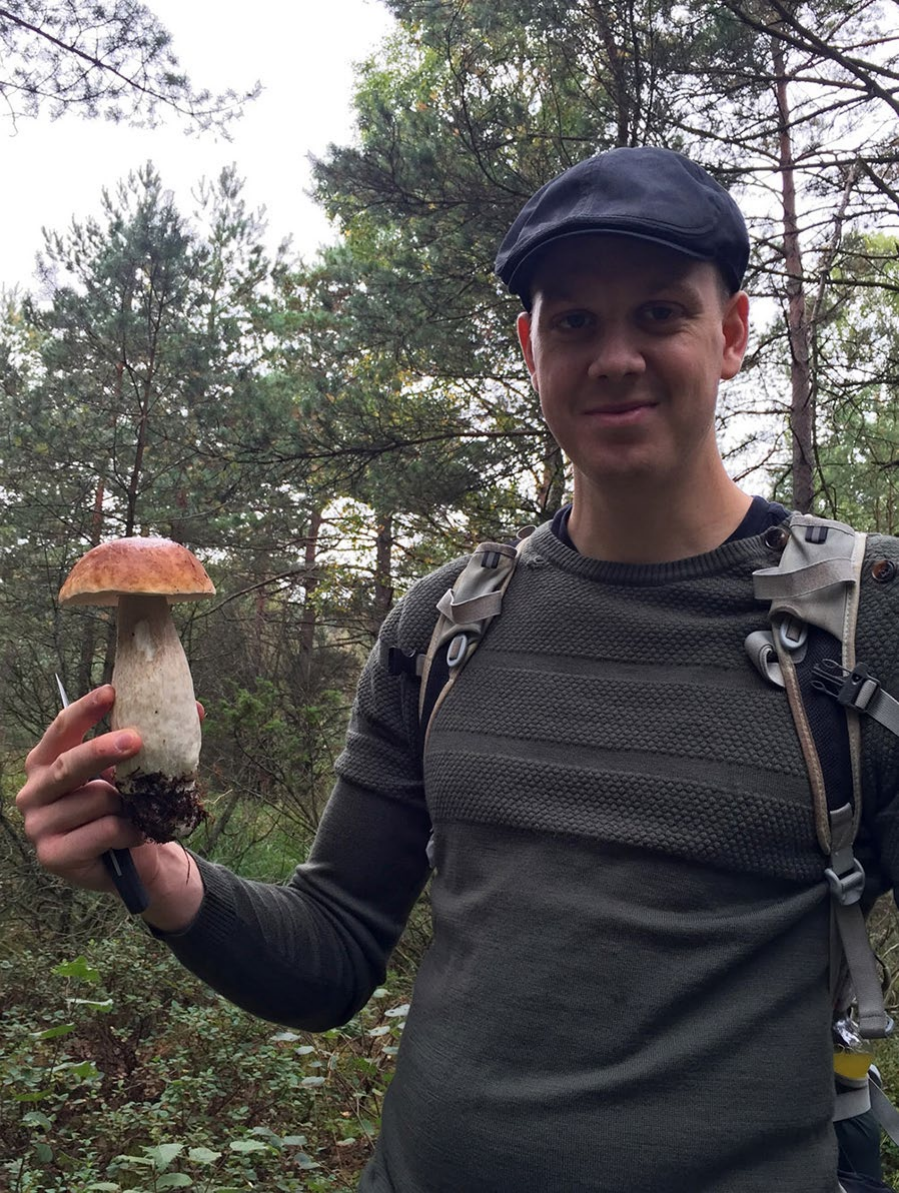
Jens during a day trip to the forests of the greater Gothenburg area—apparently being successful at picking edible mushrooms, 2016

Jens (Fig. 4) had a very intense first year with the group in Gothenburg that he had chosen for its combination of biology with informatics. It was the year before his girl-friend moved to Gothenburg. Being by himself in the beginning meant that he “could just give the project all I had”. The fantastic research environment he found in Sweden was exciting and working intensely was therefore nothing he had to force himself to. Nonetheless he would not like to continue that way in the future: “Even though I really liked this intense time, I think it’s just important to also have a balance.” And having a balance could mean for him something as simple as watching a Champion’s League game together with some childhood friends on a Wednesday evening. Jens mentions another important aspect about the intensity of research work: “the fear of failing, as you have this limited time”. The need to publish papers, as otherwise you cannot finish the Ph.D., is quite a common fear in the back of the mind of Ph.D. students, he says. Therefore, the motivation to work hard comes from within. “Hopefully it’s more based on your excitement to the work, which is definitely a major driver, but also to some degree that you don’t want to fail.”

**Fig. 5:**
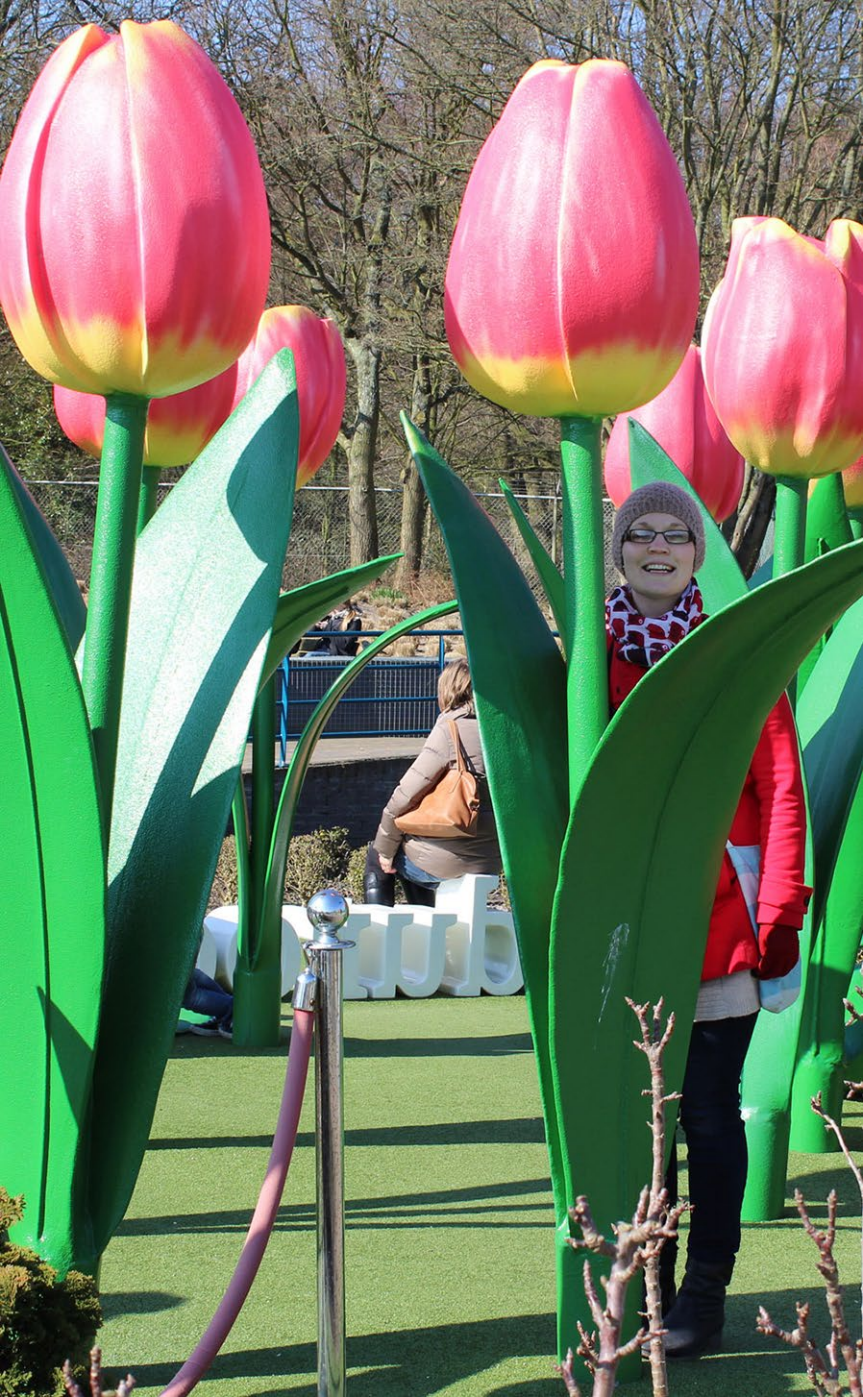
Yvonne in the Madurodam themepark in The Hague, picture taken by her mother (© Marianne Nygård) during a visit of Yvonne’s parents in 2016

Even if there are intense work periods, Yvonne (Fig. 5) tries to make enough time also for leisure activities and meeting friends and family. “I like to think that I work very systematically and organized. And all my spare time and all my sports activities are also in my calendar.” She agrees that sports are the best thing you can do to rejuvenate your energy, a common view among all the interviewed QuantFung fellows. Another favourite pastime for Yvonne is being in nature. Asking her about what she did in nature in the Netherlands she is just laughing, “That is what I missed most in the Netherlands - nature - there is not much nature around.” When the subject of the blurred border between work and private life comes up she quickly replies “There is no border for me.” and continues: “Of course it’s problematic because it means that a lot of weekends I spend working, but I do it because I enjoy my work and want to push forward.”

### Results and revelations

The outcome of an intense project time usually is a diverse one. With respect to QuantFung, for one there are measurable, scientific results—as everybody was hoping for, even though all the fellows were very much aware of the nature of research, i.e. “not all the reasonable approaches give the results as expected” (Min Jin). Then there is also the personal experience gained as a researcher when exposed to a new work environment and to a new lab with different opportunities to pursue the respective project goals. We asked the QuantFung fellows about results and revelations, about personal changes they noticed, as well as for their perspective on fungi after being immersed in this field of research for a substantial amount of time.

Sietske describes a measurable outcome that was very satisfying for her, and is an example of why and how important it is to work together with researchers with different expertise. “There was one fungus that produces a secondary metabolite, calbistrin, that was found to have an anti-cancer activity … We wanted to identify the genes encoding the biosynthetic pathway for formation of calbistrin. For this we decided to sequence the genome of the fungus in which Jens [from the QuantFung group in Goteborg] had the leading role. I analysed the structure of the compound to predict which enzymatic activities would likely be involved in the formation of the secondary metabolite and using these predictions I compared the genome sequences of three species that all produce this secondary metabolite, to predict which genes could be essential for its formation. Then we had an idea of what the gene cluster could be and Carsten [from the QuantFung group in Groningen] deleted several of the genes with the CRISPR/Cas9 based method that they had developed in the QuantFung team in Groningen. Fortunately, I saw that deletion of the genes resulted in mutant strains that did not produce calbistrin anymore, showing the power of combining all these different techniques The knowledge gained in this project could lead to the development of metabolic engineering strategies for improving calbistrin yields in future cell factories.” Sietske is convinced that “doing research changes your personality because it is so unpredictable with respect to the outcome. There are so many things not working and every time you have to tell yourself: Next time it might work, okay, there we go again” and that she “can deal with that better, after these three years. If things don’t work out - it doesn’t matter, we will try to find a different way.” Was her perception of the fungal world changed throughout the years? Yes. “It is really fascinating how they produce these secondary metabolites. … That they have these clusters of genes in the genome that all encode for enzymes to work together for making these secondary metabolites. They are so interesting because they’re all so different and we don’t actually know what these secondary metabolites are doing in nature, why they are there.”

One of Carsten’s focal points with his QuantFung group in Groningen was to develop new synthetic biology tools that can be applied to fungi. The group was also involved in quantitative biology. That was something he enjoyed and was a point of pride, as this work went beyond descriptive research to generate usable facts. Another measurable outcome was the first paper he published, which Carsten describes as follows: “We established the CRISPR-Cas9 tool in two different ways in our fungus. There were papers about this tool working in fungi before, but we showed a new way where we brought the protein into the cell directly, without it being expressed by a plasmid. It’s a method that can accelerate the process. Additionally, I was able to show that it works the same with one of the other QuantFung fellows’ fungi [Sietske at DTU]. It was very satisfying to see that it’s not just a one-time-wonder I discovered but a method that also works reliably with a different fungus. On top, it was a method helpful for my own research experiments, as it saved a lot of time.” What did this kind of collaboration do with Carsten? “I learnt how much it matters on a social level to really find a way of collaboration. I think in the beginning I was very naïve, I thought it would work automatically and we would get along just because of the great and common goal. Then I was surprised to see how much the human factor mattered, despite the common goal. The individual aspect must not be underestimated also when specialists are expected to work together. It’s something I intend to improve for myself, to be better at team-building.” And: “I still underestimated the diversity of fungi. I was specifically working with one organism. However, I think one really should review the kingdom of fungi as broad as possible to seek after useful molecules.”

Jens’ field is bioinformatics. Therefore, you won’t find him in the lab working with fungi, as he sits in front of the computer most of his work day. For the QuantFung project he was looking at genome data from various fungi. Studying these masses of genetic information led to an outcome he was very happy about: “I ended up publishing a paper in a journal called Nature Microbiology [8]. As I am just a Ph.D. student, I don’t have a huge amount of papers yet but I ended up getting something quite impactful out of the work we did, out of all these work hours spent. That was super nice. That particular paper may also help me in the future. As for the biggest revelation - how conserved everything is in life. What I find fascinating about this biology is the similarity between all species. You can take any genome of any species - if it’s a human, a bacterium or if it’s a filamentous fungus - and they actually look quite similar from my point of view. I think this “conservedness” of biology is what I find most fantastic and what I realized during my Ph.D. studies. That’s, of course, a little bit “nerdy” but I think that holds true and is really fascinating to me.”

In Berlin, Min Jin was looking for global transcription factors that regulate secondary metabolite gene expression in *A. niger*. From meta-analyses of transcriptomic data she predicted regulators in silico and verified their role in secondary metabolism by generating mutant strains. Even though she is still finalizing the data and preparing the paper, this discovery already makes Min Jin very happy as an outcome, as she describes: “I was afraid that two selected transcription factors maybe not be true regulators of secondary metabolism, so I was still a bit doubtful about our candidate genes. But when I observed colour changes of the cultivation media of the mutant strains and also sclerotia formation induced by overexpression of one transcription factor, it was the moment that I was very happy. A colour change suggests that some secondary metabolites were produced and also sclerotia formation is not a very common phenomenon with *Aspergillus niger*. Of course, I was very excited to see that.” And her perspective on fungi? “At the beginning I thought, oh, *Aspergillus niger* is very interesting, whereas now I realize that many filamentous fungi are attractive to be studied.”

Yvonne was working for QuantFung in part at the University of Groningen and for half the time she had a secondment with DSM, where she was employed by one of the project’s industrial partners. In her QuantFung project they were developing CRISPR gene editing tools and also expression systems. The results are partly published [7], which is a measurable outcome. New methodologies developed during the QuantFung project are now routinely used at the University of Groningen. With respect to the importance of comparable data sets for research, she says QuantFung could be a good starting point, as the fellows got to know the different procedures and methods in the labs where they were located. She carries on: “So you could take that to initiate some more standardization but it would need to be more long-term and also require commitment.” When we asked her about her relationship with fungi, she had to laugh: “Well, I think it’s a funny way of looking at it. But yes, that happens, you get to personalize your microorganism that you work with; I don’t know whether it’s always all that healthy…”.

### Better is the enemy of good

“I think it was a very successful project, in general it went well.” This is as brief as Yvonne puts her answer when asked for an overall balance of QuantFung. That it went very well is a common assessment among the fellows who participated in the interviews. All of them see the time with QuantFung as an important and substantial period for making progress in their respective fields of research. Without a shadow of a doubt it was a true gain for their future career path. However, even with a successful project there is always space for improvement. Like Carsten, one of the Ph.D. students, puts it “…that’s nothing that can be preconceived theoretically. … Such a project is basically like one of my experiments—of course you try to make as much risk assessment as possible but in the end you have to do it and see what it does in real life.” Which is why we asked the fellows, now in hindsight, about valued advantages with an ITN, about the things that went well in their view and about those aspects that were not perfect, so that one can learn from their experiences in order to make the next project an even better one.

The complementing effect of working on the same project, that is on the same fungus, with different tools yet the same goal, and communicating about it, is the first thing Sietske mentions with respect to advantages of this ITN project. During QuantFung she fully realized “… how valuable it is to work with researchers that have slightly different backgrounds and work on the same project. … You have the biology and the chemistry and the bioinformatics and try to combine all of that somehow. That is something I really enjoyed - to understand that all parts are needed to make one story out of that. It is also very nice to be able to talk with each other about the research - and truly understand each other. When you are doing your Ph.D. you get so specific about things that even with people who studied the same field, or even work in the same lab, it can be quite difficult to talk in depth about what you are doing and what your struggles are. But when you’re working on the same project and writing papers together, then you can discuss in depth about ‘where do we want to go’ and ‘what do we need’. … And the third thing for me: It is also just fun and nice to work together.”

Carsten “can recommend such an ITN to any young scientist, I certainly would do that again, absolutely. It really broadens the mind when you can go somewhere else and don’t stay where you are at home. That’s an important aspect. It was a great opportunity for networking, as I don’t know whether I would have been able to organize such an access to other colleagues. You may be able to meet somebody occasionally during a conference but it is not that kind of access that was provided through the meetings we had with QuantFung.” Where would be potential for improvement? “It would be good to start and extend the networking earlier. It’s just so important to be capable of teamwork in science.”

Jens found the overall balance in general a good one. “I would recommend everybody joining an ITN. Being connected with other research groups within Europe has been a real advantage. I really enjoyed that I basically could come to all the meetings and talk to some of the greatest experts in the field. All the issues I had since the last meeting, I can take up with some people who are the best to talk to about that. This connection for sharing knowledge was great.” What was good and helpful within QuantFung for your project? “What has been working very well for me was that I had a very strong collaboration since the beginning of the project with partners at DTU in Denmark [Sietske’s QuantFung group], where we’ve been working really closely together. I was doing a lot of the computational analysis and they were doing the experimental work. With Sietske, you can say both of our entire Ph.D. projects were totally intertwined, which has been very nice. My research group [in Gothenburg] has not been working that much with the filamentous fungi but with the bioinformatics techniques. … so I had that place in Sweden where I could discuss the methodology and so on, while in QuantFung I had another group of people where I could share and discuss more biological problems. And that kind of interplay has been working very well for me.”

For Min Jin the main advantage of the ITN was “to meet the different people regularly. The network an ITN provides the “human infrastructure” for discussing ideas and questions with other researchers. So that you are not working isolated by yourself all the time but regularly have an exchange - and having this possibility always available. … I think it worked very well for me and also for my family. I sure would join such a project again.” And how about potentials for improvement? “Maybe to be better organized from the very beginning. When starting as a Ph.D. or a postdoc, it’s always good to have plan, even though we don’t know many things in the beginning…”.

For Yvonne, “the network and the collaboration is the most valuable aspect for me. It was good for building up a network for your future research career. Also, it was very valuable to spend time both in the industry and at the university. For me the collaboration worked very well but for future projects there also should be some flexibility. Because it doesn’t fit everybody. It was somewhat difficult for me to move to the other part of the Netherlands [for the DSM secondment]. It’s all these practical issues that are quite challenging.”

### Continuation and prospects

Carsten will be returning to Germany after the QuantFung project. He wants to apply for a job in biotechnology in the vicinity of his hometown Potsdam. Besides being reunited with his girl-friend, what is he looking forward to when he is back home? “My grandmother has a garden in Potsdam; as a pastime I actually would like to do again some ‘farming’, to watch how things grow that I planted.” With respect to his future work, Carsten would like to continue to work with fungi. In general, the wasteful use of resources in our affluent society makes him very concerned, which is why the ecological niche for biotechnology is a lot on his mind, like fungal-based materials. “I am truly impressed by start-up companies such as Ecovative and Mycoworks who turn by-products like leftovers from cereal processing into useful, biodegradable products such as packaging materials and animal-free leather.” Though, he also stresses that “Biotechnology of the 21st century is going to be very radical when fully applied to replace an existing product. When I think about the future of synthetic biology and its possibilities, I am at first very excited about it. But then you should also realize that one need to pay price for change: it could become a competition to established industries and their products—and in the end, this poses a direct competition for human labour itself.“.

Sietske is hoping to find a research position in Tromsø so that she can continue with her work after the move from Denmark to Norway. In Tromsø there is a focus on secondary metabolites and enzymes from marine organisms, as marine organisms often produce bioactive secondary metabolites and enzymes that are active under cold conditions. She would like to join one of the research groups working on that and apply the skills she learnt from working with fungi. In every case she wants to continue with the topic of industrial microbiology. And another thing matters to her: “What I really would like to see in the next project is working with other researchers and their different perspectives. I like approaching research in an interdisciplinary way, as I did with QuantFung where I occupied myself with a lot of different aspects. …I could see me working in a project like that again.”

Min Jin is going to stay at the TU Berlin, where she was working during QuantFung. She already started a private-public collaboration project working with another filamentous fungus. She says: “It is exciting to work with different fungi to see some similarity and also differences.” Min Jin is preparing a grant proposal for a follow-up project based on the QuantFung results. Obviously, her passion for that field of research continues. As for her private life, she thinks that it is comparatively easier having a family within the university than in the industry, even though it is never going to be the fixed work day from nine to five. Asked about a working hours scheme that she would imagine in a perfect world, she says the four-days work week would be her favorite: “…for having a longer weekend and especially for having one day just for myself.”

Jens will return to Denmark, but this comes with the decision of what to do after his Ph.D.. In his view, it is basically about two options: either following an academic track or a career in industry. “I am somewhat inclined to go the industrial track where I have a bit more defined work hours.” he says. Jens has a lot of positive to say about academia and a lot of ideas for new projects to do after his Ph.D.. But, “With academia it’s just so hard because the thing about working as a professor is that it is so competitive; so if you are just going home early and you don’t get your papers and you don’t get your grants then you don’t have any Ph.D. students—and it’s just this vicious circle.” On the other hand he thinks it’s tempting to be in an academic environment where you are not as predefined in your research as you may encounter in the industry where the need to produce a specific product is prevailing. With respect to weighing the options he continues: “I have former colleagues who went straight to industry after their Ph.D. and then they thought: ‘that’s too strict’. Then they went to academia but realized that’s also tough. It can be hard to choose what is exactly good for you. Maybe you should end up being your own boss by starting some company based on secondary metabolites and filamentous fungi; that could also be a good idea. … That’s something I dream about. Let’s see. It could be amazing, I think.”

Yvonne already moved from the Netherlands to Sweden for a position as assistant professor at the Chalmers University of Technology in Gothenburg (one of the QuantFung partner universities). The focus of her research has now shifted from the quest for secondary metabolites to produce antibiotics to the production of fuels and chemicals, where for example the substitution of fossil fuels is one of the goals. She says both topics are really interesting and that it really matters to her that the research she does potentially has an application. In that respect her view is a very pragmatic one. “If I would have happened to study solar energy then probably I would be all into developing solar panels because that’s also important. I want to do meaningful work and at the moment this is the meaningful work that I do. … I think being a scientist is a privilege. I am interested in how things work and I daily get to figure out how things work. I think it’s a lot of fun.”

## The QuantFung legacy

The interdisciplinary research and training programme of QuantFung ensured that the fellows became well qualified with a broad spectrum of expertise in the field of fungal systems and synthetic biology. They are now equipped with skills and knowledge to awaken the natural product reservoir of filamentous fungi, to overproduce compounds of interest and to publish their work in peer-reviewed high impact journals. The collaboration of different academic and industrial groups led to researchers with experience of public and private work cultures and an understanding of the wider and commercial potential of their work.

For the life sciences and especially biotechnology it is essential—like a *conditio sine qua non*—to work together with each other. And this is what the QuantFung fellows have learnt. They never hesitated to ask each other because they were peers; there were no obstacles by hierarchy, they all communicated on eye level with each other. The fellows are thus much more self-confident after these years with QuantFung, as well as they are more articulate profound in scientific debates. They are not intimidated by questions anymore. Instead of evading questions they now come up with ideas of how to handle an issue, which is an important outcome of the constant communication process among the QuantFung fellows. They are also way more active asking colleagues during presentations, as they now have the courage and the experience to do that. These young scientists think along with their peers.

Another remarkable progress is the way these young scientists handle criticism. Criticism now is perceived as something positive among the fellows that underwent the QuantFung years. They understand that critical questions are nothing negative but see these questions as pieces of advice to think about for further improvement of their work. The QuantFung fellows thus became more mature scientists who grasp critical questions as part of the scientific discourse. Their next step would be to explain and picture the research work for the general public, something which they hopefully will learn during their next career steps. All things considered, ITNs such as QuantFung are ideal training networks for the new generation of (fungal) scientists. Here they can learn how to share scientific know-how and research infrastructures and how to turn research results into new technologies and products. Furthermore, they experience hands-on the importance of the human factor and of cultural exchange for unlocking their own capacities and talents. The essential assets of ITNs are indeed a very good way to advance science and enable young scientists to find their paths into a truly meaningful work life.

## Everything perfect then?

No, unfortunately not. As indicated by some of the fellows in their interviews, the job of a scientist and the career path in academia is not as attractive as it used to be. Not because one can make more money in other jobs—this is often not as relevant for students and established scientists alike, as they are intrinsically motivated and gain satisfaction from their daily work. It is because the journey into this professional future is such an unsecure one. The employment situation in academia is particularly vulnerable and one has to face the continuous challenge to get funding for new projects. This is why professors are constantly “swamped and work like mad”, to the effect that they are no longer perceived as role models for their students. Recently, the head of the Department of Experimental Neurology at the Charité Berlin, Ulrich Dirnagl, gave a glimpse of what a regular Saturday is like for him: Having to read nine project proposals of 50 pages each, plus working on a grant proposal himself. He also points out that even under the best of circumstances the chance of getting a proposal granted is 50%; in the recent past that number is closer to a mere 10%. If turned down, all the resources that went in the application process are basically wasted [10]. When looking at the success rate in ITN applications, that number even dropped to 7% (Table 2). A substantial part of scientist’s resources is thus burnt: All the work invested in a complex ITN application that can easily take up 2–3 months, including the creation of a network, the communication with potential partners and the actual grant writing which comes easily close to 50–60 pages. All of this is wasted capacity when the proposal is not successful in the end. In fact, it can be considered true economic damage, as so many work hours rendered by highly qualified experts are simply lost.

**Table 2:** Chances of success for getting an ITN proposal granted

Year of evaluation	Budget (Mio €)	No. of proposals submitted	No. of proposals granted	Success rate (%)
2010	243	900	92	10.4
2011	318	863	63	7.4
2012	423	1022	128	12.6
2013	470	1175	150	12.9
2014	405	1161	121	10.5
2015	370	1566	106	6.8
2016	370	1611	109	7.0
2017	370	1437	98	6.8

How can we make science and research more efficient? One suggestion for a new modus operandi to finance research in academia would be granting the funds in retrospect. This is a complete change of the current models where proposals are evaluated in foresight, proposals that are consequently rather promises. How could retrospect funding work? Here’s a possible scenario. University departments receive a particular budget enabling them to do teaching *and* research. After a period of 3–4 years there will be the evaluation of what is achieved with the provided funds—e.g. how many (under)graduate students defended there thesis (BSc MSc, Ph.D.) and how many publications and patents were gained. This assessment then is the base for funding for the next 3–4 years. That means the core funding may vary, according to the performance and achievements, and scientists would not thus need to spend most of their time on grant writing with insecure outcome but on doing research. Even years with a somewhat lower funding could be used for the proper preparation of new projects. Another advantage of an assessment in retrospect would be that institutions are not getting generous funds only because they have by chance somebody in their employ who is brilliant at writing grants. Funding then would rather be given to the ones demonstrated to bring the best of results.

Another suggestion, particularly for ITNs, would be the implementation of so-called two-stage proposals. Applicants write at first a project sketch that may consist of 2–3 pages only. Here they briefly outline the intended application’s concept, mention potential partners and the coordinator’s CV is added, for proving there is the experience to realize such a project. In 2017, about 1400 complete ITN applications were submitted; each of them containing 50–60 pages. If these applications were reduced to 2–3 pages project sketches and that would be taken as base for the reviewer panel to decide who is being invited to submit the full application—it clearly would make the application/funding process and thus science more efficient. Conceivable is a rate of 300 of the original 1400 submissions being invited to write full proposals with a chance to get one granted being about 30%. In that case it also would give the reviewer panel sufficient time to evaluate full proposals thoroughly. This system is in place in Germany, e.g. for calls from the Federal Ministry of Education and Research (BMBF).

Science needs its path! True innovation is often only possible at the margins of different scientific disciplines. But they must, at first, come together and get to know each other; they must comprehend how the other ones are working, what their methods are. Out of the 4 years with QuantFung, the real time for project work was 3 years for Ph.D. students and only 2 years for the post-docs. After these 2–3 years, the QuantFung fellows now have a much better idea of what needs to be considered when one intends to find and overproduce new secondary metabolites from fungi, how important the merge of different disciplines is and have achieved their first promising results. However, QuantFung as a consortium is still far away from actually having produced new classes of antibiotics, although industrial partners for the full pipeline to develop such products were within that network. Clearly, 2–3 years of research are not enough to really accomplish such a challenging mission. It is worth remembering that it was well over a decade between the discovery of penicillin and effective production [11]. This is why we propose that the EU consider applications for follow-up ITN projects. They should be limited and awarded in case of outstanding scientific achievements of an ITN consortium.

## Conclusions

**Fig. 6:**
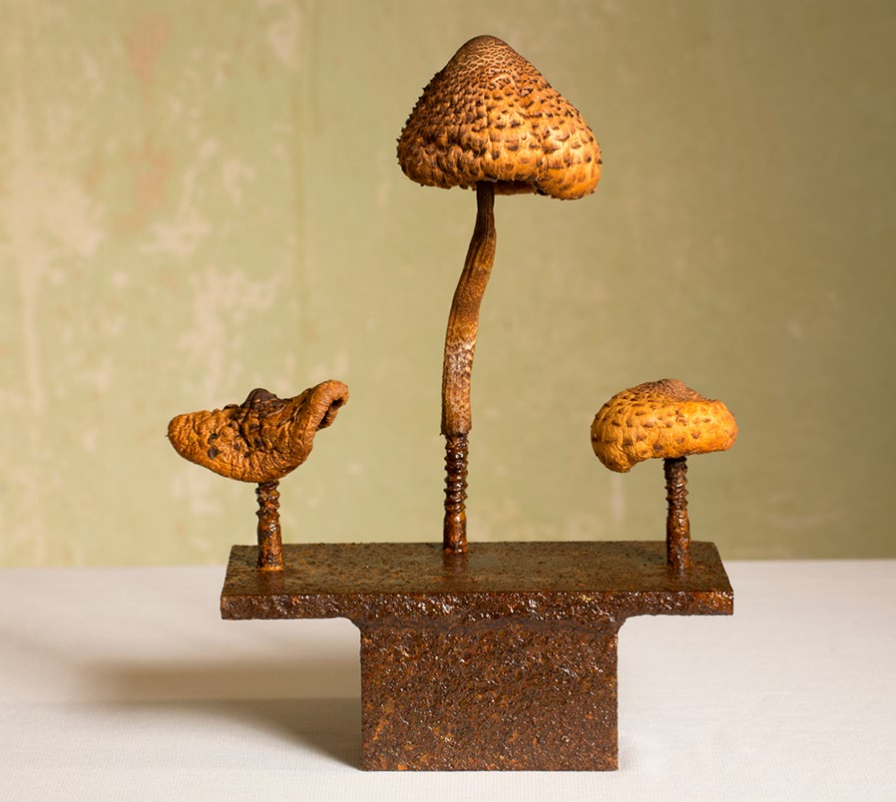
Champi(gn)ons, V. meer, 2017. Parasol mushroom, iron stand, shellac, rust (Reproduced with permission from [13])

The QuantFung participants were so immersed in the field of fungi, got so intrigued, that actually a number of fellows will continue doing research work with these organisms. Another QuantFung post-doc, Danielle Troppens, became a passionate blogger on the world of fungi. She regularly reports on findings that are curious and fascinating [12]. And as for QuantFung’s coordinator, Vera Meyer, another effect came along with her work for the QuantFung project (Fig. 6). “Fungi turned out to be not only objects of scientific study for me. I also see and use them as art objects. It is an opportunity to convey the work that we do for the general public, to get them interested. I don’t see these fields separated, science and art. Especially art for me is a means of communication. Since I have seen fungi so many times under the microscope, they became also aesthetically fascinating creatures for me. Fungi are champions for me, and it really intrigues me how to use art to give our science, the science around fungi, a face.”

ITNs represent one of the best funding instruments the EU is offering. They provide an ideal framework for international exchange and for building up a vital and innovative research environment where knowledge, state-of-the-art technologies and the most modern equipment can be shared. All of which is what is needed to train our next generation of high-calibre scientists, to foster their creativity and to jointly come to new scientific breakthroughs. How beneficial it is to have such a network was strikingly proven by the success that QuantFung had on many levels. There are the measurable results and there is the fact that 15 young researchers are now exquisitely prepared for a future career in biotechnology. The fellows are well qualified with a broad portfolio of skills and practical experience of cross sector working; this will make them strong candidates for future employers and underpin their career progression. Importantly, taking responsibility for their own development has instilled in them at an early stage the importance of continuous professional development. Such high quality and highly competitive candidates are needed in both the public and private sectors. Although academia may lose great talents, they have excellent chances to take up industry posts.

“Antibiotic resistance is one of the biggest threats to global health, food security, and development today.” [14]. This is the first sentence of the World Health Organization’s internet fact sheet addressing the crisis of more and more frequently failing antibiotic medication. Which leaves nobody in doubt about the problem’s dimension. There are ambitious researchers in academia who eagerly want to contribute to the problem’s solution. But they should be given the opportunity to pursue their respective work lives in a way that leaves them space to breathe, instead of keeping them in the uncertainty of perpetual-temporary contracts and in the frenzy of constant grant writing. A “Vita activa” in Hannah Arendt’s view means a life spent reaching up to higher goals. Advancing the deeper understanding of fascinating organisms like filamentous fungi for the sake of human health is certainly such a goal.

## References

[1] Arendt H (2005). Vita activa oder Vom tätigen Leben.

[2] Cooper MA, Shlaes D (2011). Fix the antibiotics pipeline. Nature.

[3] Büttel Z, Díaz R, Dirnberger B, Flak M, Grijseels S, Kwon MJ, Nielsen JCF, Nygård Y, Phule P, Pohl C, Prigent S, Randelovic M, Schütze T, Troppens D, Viggiano A (2015). Unlocking the potential of fungi: the QuantFung project. Fungal Biol Biotechnol.

[4] http://mikrobiologie.eurofung.tu-berlin.de.

[5] Takano E, Bovenberg RA, Breitling R (2012). A turning point for natural product discovery: ESF-EMBO research conference: synthetic biology of antibiotic production. Mol Microbiol.

[6] http://intern.mikrobiologie.tu-berlin.de.

[7] Pohl C, Kiel JA, Driessen AJ, Bovenberg RA, Nygård Y (2016). CRISPR/Cas9 Based Genome Editing of *Penicillium chrysogenum*. ACS Synth Biol.

[8] Nielsen JC, Grijseels S, Prigent S, Ji B, Dainat J, Nielsen KF, Frisvad JC, Workman M, Nielsen J (2017). Global analysis of biosynthetic gene clusters reveals vast potential of secondary metabolite production in *Penicillium* species. Nat Microbiol.

[9] Meyer V, Schütze T (2017). Polycistronic gene expression in *Aspergillus niger*. Microb Cell Factories.

[10] Ulrich Dirnagl. Werden Sie Forschungsförderer. In: Laborjournal 2017-06, pp 22–23.

[11] https://www.pfizer.com/about/history/all.

[12] www.microbe-scope.com.

[13] www.v-meer.de.

[14] www.who.int/mediacentre/factsheets/antibiotic-resistance/en/.

[15] www.menschenfotograf.de/en/workspace-canada-2.

